# Unveiling E2F4, TEAD1 and AP-1 as regulatory transcription factors of the replicative senescence program by multi-omics analysis

**DOI:** 10.1007/s13238-021-00894-z

**Published:** 2022-01-12

**Authors:** Yuting Wang, Liping Liu, Yifan Song, Xiaojie Yu, Hongkui Deng

**Affiliations:** 1 School of Basic Medical Sciences, State Key Laboratory of Natural and Biomimetic Drugs, Peking University, Beijing, 100191 China; 2 The MOE Key Laboratory of Cell Proliferation and Differentiation, College of Life Sciences, Peking-Tsinghua Center for Life Sciences, Peking University, Beijing, 100871 China; 3 State Key Laboratory of Chemical Oncogenomics, School of Chemical Biology and Biotechnology, Peking University Shenzhen Graduate School, Shenzhen, 518055 China

**Keywords:** transcription factor, senescence, multi-omics

## Abstract

Senescence, a stable state of growth arrest, affects many physiological and pathophysiological processes, especially aging. Previous work has indicated that transcription factors (TFs) play a role in regulating senescence. However, a systematic study of regulatory TFs during replicative senescence (RS) using multi-omics analysis is still lacking. Here, we generated time-resolved RNA-seq, reduced representation bisulfite sequencing (RRBS) and ATAC-seq datasets during RS of mouse skin fibroblasts, which demonstrated that an enhanced inflammatory response and reduced proliferative capacity were the main characteristics of RS in both the transcriptome and epigenome. Through integrative analysis and genetic manipulations, we found that transcription factors E2F4, TEAD1 and AP-1 are key regulators of RS. Overexpression of *E2f4* improved cellular proliferative capacity, attenuated SA-β-Gal activity and changed RS-associated differentially methylated sites (DMSs). Moreover, knockdown of *Tead1* attenuated SA-β-Gal activity and partially altered the RS-associated transcriptome. In addition, knockdown of *Atf3*, one member of AP-1 superfamily TFs, reduced *Cdkn2a* (*p16*) expression in pre-senescent fibroblasts. Taken together, the results of this study identified transcription factors regulating the senescence program through multi-omics analysis, providing potential therapeutic targets for anti-aging.

**Supplementary Information:**

The online version contains supplementary material available at 10.1007/s13238-021-00894-z.

## INTRODUCTION

Senescence is known as a stable state of cell cycle arrest, characterized by elevated *Cdkn2a* (*p16*) and *Cdkn1a* (*p21*) expression, the senescence-associated secretory phenotype (SASP) and epigenetic changes (Hernandez-Segura et al., 2018). Senescence plays important roles in many physiological and pathophysiological processes, especially aging (van Deursen, 2014). Previous work by our group and others showed that clearance of senescent cells attenuated aging-associated phenotypes *in vivo* (Baker et al., 2016; Cai et al., 2020). In contrast, transplantation of small numbers of senescent cells into recipients induced aging-related phenotypes, spread cellular senescence to host tissues and increased mortality (Xu et al., 2018). These results demonstrated that cellular senescence play crucial roles in organismal aging and aging-related diseases.

Cellular senescence can be induced by various stimuli *in vitro*, including proliferation, oncogenes, radiation and chemicals (van Deursen, 2014; Hernandez-Segura et al., 2018). However, senescence induced by different stimuli may have different mechanisms, and distinct transcriptional and epigenetic signatures (Nelson et al., 2014; Sanokawa-Akakura et al., 2019). Previous work comparing the transcriptional profiles of stem cell aging *in vivo* and RS *in vitro*, demonstrated that stem and progenitor cells *in vivo* undergo a process similar to RS (Wagner et al., 2009). In addition, the genetic signatures of upregulated genes associated with RS was found to significantly overlap with that of aging *in vivo*, highlighting the similarity between RS and aging *in vivo* (Avelar et al., 2020). Moreover, RS and aging *in vivo* are both associated with highly reproducible epigenetic changes, especially changes in DNA methylation (Wagner et al., 2016). Conserved aging-associated DNA methylation changes were observed in RS and aging *in vivo* (Minteer et al., 2020), indicating that RS and aging *in vivo* may be governed by the same conserved mechanisms (Wagner et al., 2016). These results highlight the important role of RS in aging, suggesting that the study of mechanisms and regulatory networks associated with RS could provide potential targets for anti-aging interventions.

RS is characterized by growth arrest, shortened telomeres, elevated SA-β-Gal activity and SASP (Coppé et al., 2008; Purcell et al., 2014; Hernandez-Segura et al., 2018). In addition, RS is accompanied by widespread transcriptional and epigenetic changes (Purcell et al., 2014; Hänzelmann et al., 2015; Chan et al., 2021; Zhang et al., 2021). However, the mechanisms and regulators underlying the phenotypes of RS remain unclear. Recently, AP-1 family transcription factors were found to play an important role in regulating the senescence program, and knockdown of AP-1 was found to attenuate part of senescence phenotypes (Martínez-Zamudio et al., 2020; Zhang et al., 2021), which highlights the regulatory roles of TFs in RS. Thus, a systemic study of the regulatory TFs underlying RS could provide insight into the mechanisms and provide direction for the development of anti-senescence interventions.

Here, to systematically analyze changes in the transcriptome and epigenome associated with RS, and identify the regulatory TFs underlying RS, we employed integrative and time-series analysis based on RNA-seq, RRBS and ATAC-seq of mouse skin fibroblasts. The results demonstrated that an enhanced inflammatory response in late senescence and a gradual decline in replicative capacity were significant signatures of RS in the transcriptome and epigenome. In addition, by using bioinformatic analysis and genetic manipulations, we identified E2F4, TEAD1 and AP-1 as regulatory TFs of RS that influenced the phenotypes of RS. Taken together, our results reveal regulatory TFs underlying RS, which provides novel targets for senescence and aging interventions.

## RESULTS

### Transcriptional dynamics and signatures of replicative senescence in mouse skin fibroblasts

**Figure 1:**
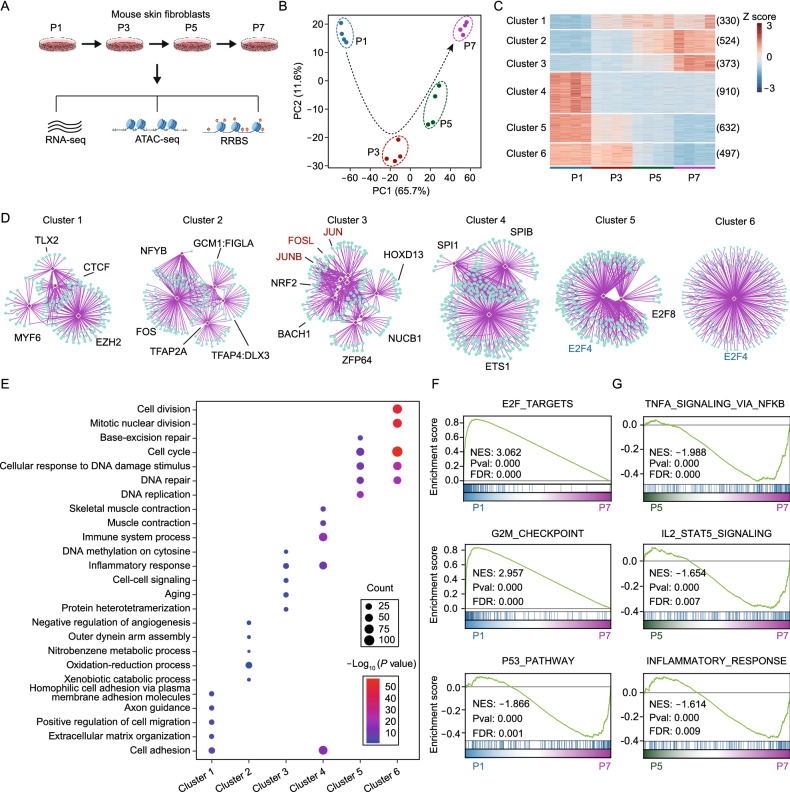
**Transcriptional signatures and regulators of replicative senescence**. (A) Schematic outlining the design of multi-omics analysis of cellular replicative senescence. Skin fibroblasts from 2-month-old mice were used, and samples were collected at P1, P3, P5 and P7 for RNA-seq, ATAC-seq and RRBS. (B) PCA of the transcriptome of skin fibroblast during RS. P1 (blue), P3 (dark red), P5 (dark green) and P7 (purple), (*n* = 4). (C) Heatmap showing 6 major modules of differentially expressed genes during RS. Genes that differentially expressed in any group when compared with P1 are shown (adjusted *P*-value < 0.05 and fold change > 2). The number of genes within each cluster is given in parentheses, (*n* = 4). (D) Transcription factor enrichment analysis based on the genes from each cluster. Only the top significantly enriched TFs are shown. AP-1 family TFs and E2F4 are highlighted. The analysis was performed with RcisTarget. (E) Selected biological process GO terms enriched in each gene cluster, with the top 5 most significantly enriched terms shown. The size of each point represents the gene count in the GO term, and the color of each point represents the significance of the enrichment. (F) GSEA showing cell cycle-related terms based on the transcriptome of P1 versus P7, (*n* = 4). (G) GSEA showing inflammatory response-related terms based on the transcriptome of P5 versus P7, (*n* = 4)

To reveal the transcriptome and epigenome signatures of RS, we employed time series analysis of mouse skin fibroblasts with RNA-seq, RRBS and ATAC-seq at 4 different passage numbers (P1, P3, P5 and P7) (Fig. 1A). We examined senescence markers in P7 cells, including EdU staining, nuclear morphology and SA-β-Gal activity, and confirmed that cells entered into replicative senescence at P7 (Fig. S1A–C). Global gene expression profiles generated using principal component analysis (PCA) of the RNA-seq data demonstrated significant transcriptome changes with passaging (Fig. 1B). To further explore differentially expressed genes during senescence, 3,266 significantly changed genes compared with P1 were selected and clustered into 6 modules using unsupervised methods (Figs. 1C and S1D).

Next, we identified potential regulatory TFs based on genes from different clusters with RcisTarget (See METHODS). Interestingly, we found that genes from cluster 3, which showed elevated expression at late senescence, were enriched with AP-1 family TFs, including JUNB, JUN and FOSL (Fig. 1D). AP-1 family TFs have been reported to play a role in the inflammatory response (Uluçkan et al., 2015; Ji et al., 2019). In contrast, genes from cluster 5 and cluster 6, whose expression gradually decreased with passaging, were enriched with E2F4 (Fig. 1D), a TF related to cell cycle regulation (Garneau et al., 2009; Hsu et al., 2019). Subsequently, we employed Gene Ontology (GO) analysis based on genes from different clusters to examine changes in biological functions during RS. In line with the TF enrichment analysis, we found that genes from cluster 3 were significantly enriched with inflammatory response and aging process-related terms. In contrast, genes from cluster 5 and cluster 6 were enriched with cell cycle-related terms (Fig. 1E).

To confirm the transcriptome signatures of RS, we employed gene set enrichment analysis (GSEA). Consistent with the GO analysis, we found that cell cycle-related pathways like G2M checkpoint and E2F targets, were significantly down-regulated with passaging. In contrast, aging-associated pathways, like P53, were up-regulated in senescent cells (Fig. 1F). Remarkably, we also found inflammation related pathways, like TNF-α signaling, IL-2 and inflammatory response, were significantly up-regulated at late senescence (Fig. 1G). In line with transcriptional analysis, SASP in culture supernatant was found to increase dramatically when cells entered into senescence (Fig. S1E), as previously reported (Coppé et al., 2008).

Collectively, these results demonstrated that RS was accompanied by significant transcriptome changes. The main characteristics of RS were an enhanced inflammatory response at late senescence and a gradual decline in replicative capacity.

### Time-resolved DNA methylome profiling to reveal RS-associated epigenetic signatures in mouse skin fibroblasts

**Figure 2:**
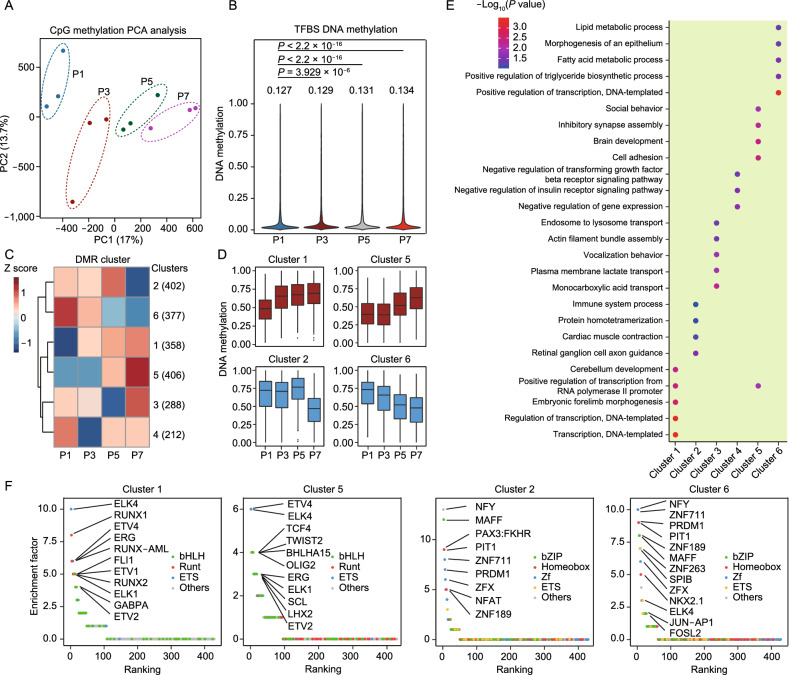
**RS-associated DNA methylome signatures in mouse skin fibroblasts**. (A) Unsupervised PCA of RRBS in skin fibroblasts during RS. P1 (blue), P3 (dark red), P5 (dark green) and P7 (purple), (*n* = 3). (B) Violin plot summarizing the distribution of DNA methylation levels of the transcription factor binding sites (TFBS) of each group. TFBS were identified by ATAC-seq. The DNA methylation level of each TFBS was estimated by averaging the methylation level of all cytosines within that TFBS. (C) Heatmap showing 6 major modules of the dynamic average methylation levels of DMRs. The number of DMRs within each modules is given in parentheses. The color indicates the average of the DNA methylation levels of all included DMRs in each module, (*n* = 3). (D) Box plot summarizing the distribution and dynamics of the DNA methylation levels of DMRs in module 2, module 6, module 1 and module 5, (*n* = 3). (E) Biological process GO term enrichment analysis based on genes related to the DMR modules, with the top 5 significantly enriched terms shown. The DMR-related genes were identified when the DMRs were located within ±5 kb around the transcription start site (TSS). (F) Ranking of motifs enriched in senescence-associated DMRs from cluster 1, cluster 5, cluster 2 and cluster 6. The colors of the points represent different TF families. The data were analyzed by one-way ANOVA (B). The error bars show the SD (D)

The epigenome plays an important role in transcriptome regulation and cell fate determination. Although some senescence-associated epigenetic signatures have been documented and used as markers of senescence, a systematic analysis of chromatin accessibility and DNA methylation changes during RS has not been performed. To study changes in DNA methylome during RS, we employed time-series RRBS analysis. PCA analysis based on RRBS showed significant changes in DNA methylation with RS (Fig. 2A). Although no obvious change in distribution of DNA methylation across the entire genome was observed, DNA methylation at TFs binding sites (TFBSs) gradually increased with passaging (Figs. S2A and 2B), suggesting decreased chromatin accessibility at TFBSs and reduced TF binding activity with RS.

Next, we identified differentially methylated regions (DMRs) with passaging using DSS (Feng et al., 2014) and divided them into 6 clusters with unsupervised methods (Fig. 2C). Among these DMRs, the hypomethylated (cluster 2 and 6) and hypermethylated (cluster 1 and 5) DMRs with monotonically changed DNA methylation were identified as RS-associated DMRs (Fig. 2D). To identify which biological processes were influenced by RS-associated DMRs, we performed GO analysis based on genes related to RS-associated DMRs. For RS-associated up DMRs (cluster 1 and 5), the most enriched GO terms included transcription regulation and development-related biological processes. In contrast, for RS-associated down DMRs (cluster 2 and 6), the most enriched GO terms included metabolic process and immune system process (Fig. 2E), consistent with transcriptional analysis.

In addition, we also identified RS-associated differentially methylated sites (DMSs), which monotonically changed with passaging in three independent series of samples (See METHODS). In total, 77 DMSs were identified, including 38 up DMSs and 39 down DMSs (Fig. S2B). Next, we performed genomic annotation of RS-associated DMSs using HOMER. Most of RS-associated up DMSs were located at genetic related regions, like introns, exons and promoters. However, down DMSs were mainly located at intergenic regions (Fig. S2C). These results suggested that promoters and gene bodies become increasingly methylated with senescence. In line with the analysis of RS-associated down regulated genes, GO analysis based on genes related to up DMSs indicated that development-related biological processes were deactivated with RS (Fig. S2D).

Through integrative analysis of DNA methylation and chromatin accessibility, we found a significant negative correlation between DNA methylation and chromosome accessibility (Fig. S2E and S2F). Changes in DNA methylation may affect chromatin accessibility, which could influence TF binding in further. To explore the TFs affected by methylome changes with RS, we performed motif analysis based on RS-associated DMRs using HOMER (See METHODS). For RS-associated up DMRs, the most enriched motifs were from bHLH, Runt and ETS families, including ETV4, ELK4 and ERG. For RS-associated down DMRs, the most enriched motifs were from bZIP and Homeobox families, including NFY, PIT1 and AP-1, suggesting that activation of these TFs occurred during RS (Fig. 2F).

Taken together, the results of our analysis demonstrated significant methylome changes and continuously increased DNA methylation of TFBSs with passaging. Consistent with the transcriptome analysis, RS-associated DMRs and DMSs were found to be related to metabolic processes, immune processes and development, which highlighted the consistency of the transcriptome changes and methylome changes during RS.

### Chromatin accessibility dynamics regulate RS-associated transcriptional signatures

**Figure 3:**
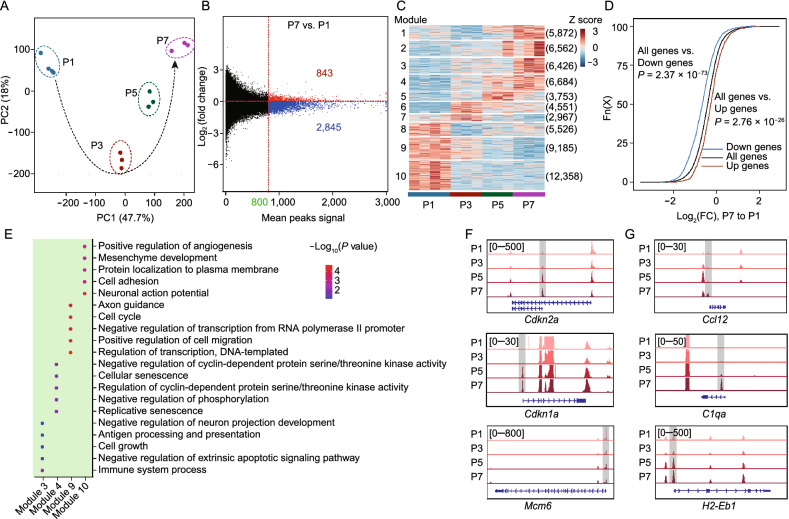
**Chromatin accessibility dynamics of replicative senescence in mouse skin fibroblasts**. (A) PCA of chromatin accessibility detected with ATAC-seq in skin fibroblasts during RS. P1 (blue), P3 (dark red), P5 (dark green) and P7 (purple), (*n* = 4 for P1 and *n* = 3 for P3, P5 and P7). (B) MA plot of senescent cells (P7) and proliferating cells (P1) showing the log2 ratios of peak accessibility versus the mean normalized read count for all atlas peaks. For peaks with a mean signal greater than 800, the numbers of peaks with open (red) and closed (blue) chromatin accessibility were calculated. (C) Heatmap showing 10 major modules of dynamic accessibility of peaks during RS. The number of peaks within each module is given in parentheses (*n* = 4 for P1 and *n* = 3 for P3, P5 and P7). (D) Cumulative distribution showing the chromatin accessibility differences between peaks related to RS-associated up-regulated genes and down-regulated genes (P7 vs P1). Peaks associated with up-regulated genes had greater accessibility when compared with all genes (*P* = 2.76 × 10^−26^). Peaks associated with down-regulated genes had lower accessibility when compared with all genes (*P* = 2.37 × 10^−73^). (E) Biological process GO term enrichment analysis based on genes related to peaks in selected modules, with only the top 5 significantly enriched terms shown. The color of each point represents the significance of the enrichment. (F and G) Browser showing chromatin accessibility changes during RS. Browser representation of normalized ATAC-seq coverage around TSS of cell cycle regulators, including *Cdkn2a*, *Cdkn1a* and *Mcm6* (F) and inflammatory response-related genes, including *Ccl12*, *C1qa* and *H2-Eb1* (G). The grey bars highlighted aging-associated peaks in promoter regions. Data were analyzed by *t-*test (D)

To reveal the chromatin accessibility dynamics of RS, we performed time-resolved ATAC-seq during passaging. PCA analysis and sample correlation analysis based on the ATAC-seq data showed significant changes in chromatin accessibility with passaging (Figs. 3A and S3A). Elevated expression of transposable elements was observed in senescence (Colombo et al., 2018; De Cecco et al., 2019; Potocki et al., 2019), suggesting increased instability of the genome. However, in our analysis, no obvious change in chromatin accessibility was observed across the entire genome with passaging. Nevertheless, for peaks with higher coverage (mean value > 800), decreased chromatin accessibility was observed with RS (Fig. 3B), which was consistent with other recent studies (Wang et al., 2018; Hernando-Herraez et al., 2019).

To further explore chromatin accessibility dynamics with passaging, we identified differentially changed peaks during RS and divided them into 10 modules using unsupervised methods (See METHODS). Importantly, open peaks from module 3 and module 4, and closed peaks from module 9 and module 10 showed monotonic changes with passaging, and were identified as RS-associated peaks (Fig. 3C), which were evenly distributed across the chromosomes (Fig. S3B). Peaks annotation demonstrated that RS-associated closed peaks were more likely to be located in promoter regions (10.51% and 2.05% for module 9 and module 10, respectively) in comparison with RS-associated open peaks (0.75% and 0.64% for module 3 and module 4, respectively) (Fig. S3C). Considering that RS was found to be associated with increased DNA methylation in TFBSs, these results might indicate decreased transcriptional activity with passaging.

The epigenetic state of promoter regions influences the expression of corresponding downstream genes (Qu et al., 2017; Wong et al., 2017). Through integrative analysis of ATAC-seq and RNA-seq, we found that the promoter accessibility of RS-associated up-regulated genes was significantly greater than that of RS-associated down-regulated genes (Fig. 3D). Next, we examined the biological processes influenced by RS-associated peaks through GO analysis of corresponding genes (See METHODS). In agreement with transcriptional analysis, peaks from module 3 were enriched with development, apoptotic signaling and immune system process. Peaks from module 4 were enriched with cellular senescence and protein kinase activity, whereas peaks from module 9 and 10 were enriched with cell cycle and cellular interaction-related processes (Fig. 3E). These results demonstrated that the transcriptional signatures of cellular senescence were associated with corresponding epigenetic changes. Moreover, up-regulation of the inflammatory response and down-regulation of proliferative capacity were found to be the main signatures of RS in both transcriptome and epigenome.

To confirm epigenetic changes underlying RS-associated transcriptional signatures, we visualized the promoter accessibility dynamics of selected genes with Integrative Genomics Viewer (IGV). For example, *Cdkn2a* (*p16*) and *Cdkn1a* (*p21*) are negative regulators of cell cycle, which are widely used as senescence markers. The IGV showed that the promoter accessibility of these two genes gradually increased with passaging. However, for cell cycle positive regulators, such as *Mcm6*, the promoter accessibility gradually decreased with passaging (Fig. 3F). In line with this, decreased promoter accessibility of most cell cycle-related genes was observed with RS (Fig. S3D and S3E). In contrast, for inflammatory response related genes, such as *Ccl12*, *C1qa* and *H2-Eb1*, the promoters showed increased chromatin accessibility with passaging (Fig. 3G).

Altogether, these results demonstrated that RS was accompanied by significant epigenetic changes, which could in further influence transcriptome. In addition, up-regulation of inflammatory response and down-regulation of proliferative capacity were two main signatures of RS in both transcriptome and epigenome.

### Identification of replicative senescence-associated regulatory TFs

**Figure 4:**
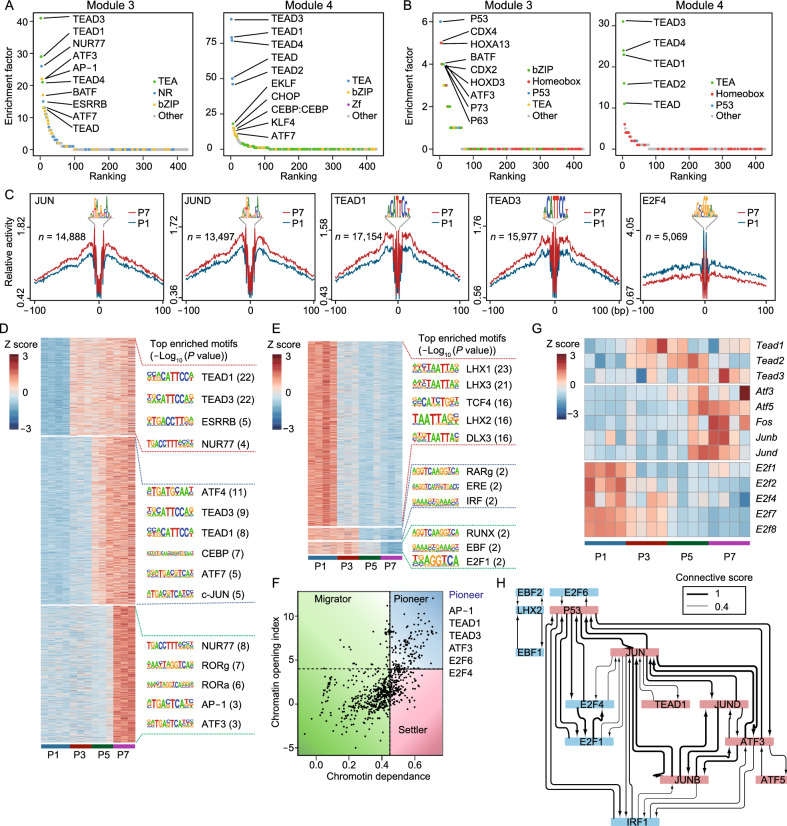
**Identification of replicative senescence-associated regulatory TFs**. (A and B) Ranking of motifs enriched in senescence-associated open peaks. The colors of the points represent different TF families. (A) All open peaks from module 3 and module 4. (B) Open peaks located at enhancer regions from module 3 and module 4. (C) Transcription factor footprinting in chromatin-accessible regions based on ATAC-seq. The mean normalized ATAC-seq coverage averaging the forward and reverse strand within 100 bp upstream and downstream of the TF motif is shown. The red line represents P7, and the blue line represents P1. (D) Heatmap showing peaks appearing in closed regions during RS. Selected enriched motifs from these peaks and the corresponding levels of enrichment significance are shown. (E) Heatmap showing peaks disappearing from open regions during replicative senescence. Selected enriched motifs from these peaks and the corresponding levels of enrichment significance are shown. (F) Classification of transcription factors based on chromatin dependence (CD) and the chromatin opening index (COI). Pioneer factors (blue box, COI > 4 and CD > 0.45), settler factors (red box, COI < 4 and CD > 0.45) and migrator factors (green box, CD < 0.45) were defined, and selected top pioneer factors are shown. (G) Heatmap showing the expression dynamics of potential regulatory TFs of RS. (H) Regulatory networks showing the connections between RS-associated TFs. TFs with pink backgrounds have increased activity during senescence, and TFs with blue backgrounds have decreased activity during senescence. The thickness of the lines between TFs indicates the connectivity score

TFs were involved in regulation of various biological processes (Spitz and Furlong, 2012). Thus, we speculated that the cellular senescence program could be regulated by specific TFs like other cellular processes. To identify RS-associated TFs, we firstly performed motif enrichment analysis based on RS-associated peaks using HOMER. Notably, RS-associated open peaks (module 3 and module 4) were enriched with TEA family TFs, like TEAD1 and TEAD3, which were also revealed in human senescent cells recently (Chan et al., 2021). In addition, peaks from module 3 were also found to be enriched with motifs from AP-1 family TFs (Fig. 4A), which was observed to activate in oncogene-induced senescence recently (Martínez-Zamudio et al., 2020). In RS-associated closed peaks (module 9 and module 10), motifs from EBF, LHX family TFs were significantly enriched (Fig. S4A).

Enhancers are specific genome regions, which influence the TF binding and expression of relatively distant genes (Spitz and Furlong, 2012). Based on H3K4me1 and H3K27ac ChIP-seq data from public database GSE117210 (Guan et al., 2020), we identified enhancer regions in senescent mouse fibroblasts (See METHODS). To further explore TFs enriched in enhancers during RS, we performed motif enrichment analysis based on ATAC-seq peaks overlapped with enhancer regions. For peaks from module 3, the most enriched TF located at enhancers was P53, a negative regulator of cell cycle in various senescence models. In addition, for peaks from module 4, the top enriched TFs located at enhancers were from TEA family, including TEAD3, TEAD1 (Fig. 4B). In contrast, for RS-associated closed peaks (cluster 9 and cluster 10), the top enriched motifs located at enhancers included EBF, ETV2 and LHX3 (Fig. S4B).

The activity changes of TFs could be reflected through relative coverage around binding sites using RGT-HINT (Li et al., 2019). Through analysis, we found that AP-1 family TFs, including cJUN and JUND, and TEA family TFs, including TEAD1 and TEAD3, showed increased activity with passaging. In contrast, E2F4 showed decreased activity with senescence (Figs. 4C, S4C and S4D).

Among TFs, pioneer factors could bind to compact chromatin regions, open these regions and recruit other factors to bind around (Iwafuchi-Doi and Zaret, 2014), which may play crucial roles in regulating the senescence program. According to the characteristics of pioneer factors, we hypothesized that newly appeared peaks during RS might be due to pioneer factors binding. Based on this hypothesis, we identified newly appeared peaks during passaging and performed motif enrichment analysis. The results demonstrated that TEA family TFs were significantly enriched in newly appeared peaks from P1 to P3. In addition, the most significantly enriched TFs in newly appeared peaks at P5 and P7 were AP-1 family TFs (Fig. 4D). We also identified peaks that disappeared during RS and performed motif enrichment analysis. Consistent with the motif analysis described above, these peaks were mainly enriched with TFs from LHX, RUNX and E2F families (Fig. 4E). As reported, pioneer factors could also be identified through calculating chromatin dependence (CD) and chromatin opening index (COI) using PIQ based on ATAC-seq (Sherwood et al., 2014). In our analysis, TFs with CD greater than 0.45 and COI greater than 4 were identified as pioneer factors. Consistent with the analysis described above, AP-1, TEAD1, TEAD3, ATF3, E2F4 and E2F6 were included in identified pioneer factors of RS (Fig. 4F).

Next, we examined changes in expression levels of RS-associated TFs during senescence. Consistent with the observed changes in TF activity, AP-1 family TFs, including *Atf3*, *Fos*, *Junb* and *Jund* showed increased expression when cells entered into late senescence. TFs from TEA family, such as *Tead1*, showed increased expression even from the early stage of passaging. In contrast, the expression of E2F family TFs, including *E2f4*, decreased gradually with passaging (Fig. 4G). In addition, regulatory networks analysis of identified RS-associated TFs showed a complicated regulatory relationship between these TFs. Interestingly, P53, a widely used senescence marker, was found to be under the common regulation of AP-1 and E2F family TFs (Fig. 4H).

Taken together, several potential regulatory TFs of RS were identified by integrative analysis of transcriptome and epigenome. Among them, TFs from TEA and AP-1 family showed increased activity and expression with passaging, like TEAD1 and ATF3. In contrast, TFs from E2F family showed decreased activity and expression, like E2F4.

### E2F4 affects RS-associated phenotypes as a regulatory TF

E2F4 was identified as a potential regulatory TF of RS in our previous analysis (Fig. 4C and 4F), which was also enriched in RS-associated down-regulated genes (Fig. 1D). In addition, conserved change in expression of E2F4 with passaging was observed in human cells after analysis of public RNA-seq data from GSE109700 (De Cecco et al., 2019) (Fig. S5A), suggesting that the regulatory role of E2F4 in senescence may be species conserved.

To further explore the regulatory roles of E2F4 in RS, we performed an integrative analysis of RNA-seq and ATAC-seq data with DiffTF (Berest et al., 2019). E2F4 was identified as a positive regulator of downstream genes (*P* < 0.001) (Fig. S5B). Notably, E2F4 CUT&Tag analysis indicated that E2F4 regulated cell cycle process during senescence (Fig. S5C), highlighting regulatory roles of E2F4 in RS.

**Figure 5:**
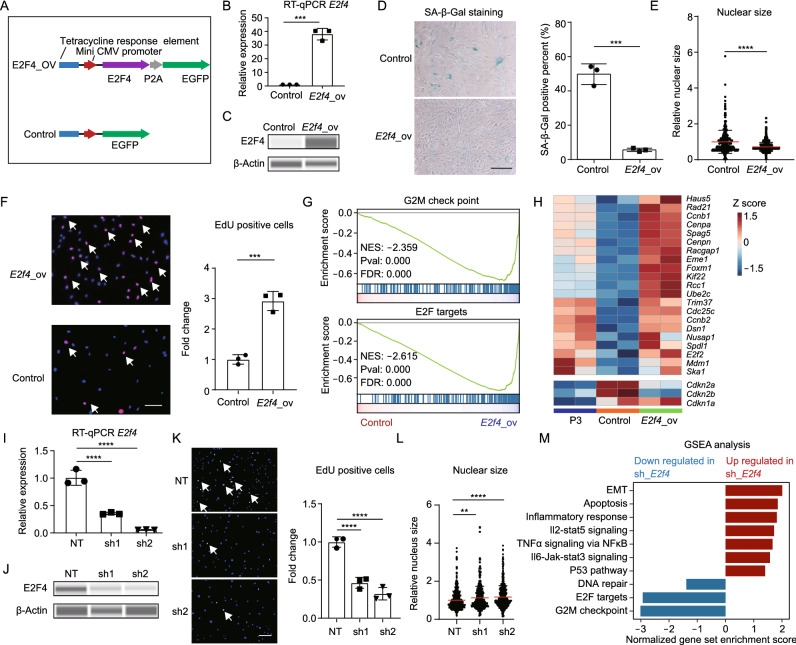
**E2F4 affects replicative senescence-associated phenotypes as a regulatory TF**. (A) Structures of plasmids used for EGFP-labeled E2F4 expression under doxycycline induction and corresponding control. (B) RT-qPCR showing the change in the *E2f4* expression level after overexpression with lentivirus, (*n* = 3). (C) Immunoblot showing the change in E2F4 protein level after overexpression. (D) Change in SA-β-Gal activity after *E2f4* overexpression. SA-β-Gal positive percentage was estimated based on 3 different views, and the points indicated the average value, (*n* = 3). Scale bar = 200 μm. (E) Scatter plot showing the nuclear size distribution after *E2f4* overexpression based on nuclear staining. Nuclear size was calculated using ImageJ, (*n* = 3). (F) EdU staining showing the change in proliferating capacity after *E2f4* overexpression. Red dots indicate EdU positive cells (white arrows), and the EdU-positive percentage was calculated with ImageJ. The positive percentage was estimated based on 5 different views, and the points indicated the average value, (*n* = 3). Scale bar = 200 μm. (G) GSEA showing changes in cell cycle-related terms after *E2f4* overexpression based on RNA-seq. (H) Heatmap showing changes in the expression levels of cell cycle-related genes, including positive regulators (up) and negative regulators (down). P3 samples were used as proliferating cell control, (*n* = 2). (I) RT-qPCR detecting the change in *E2f4* expression level after shRNA-mediated knockdown, (*n* = 3). (J) Immunoblot showing the change in E2F4 protein level after knockdown. (K) EdU staining showing the change in proliferating capacity after *E2f4* knockdown. Green dots represent EdU-positive cells (white arrows), (*n* = 3). Scale bar = 200 μm. (L) Scatter plot showing the nuclear size distribution after *E2f4* knockdown based on nuclear staining. Nuclear size was calculated using ImageJ, (*n* = 3). (M) Bar plot showing the changes of RS-associated pathways after *E2f4* knockdown through GSEA analysis. Blue bar indicates down-regulated pathways, and red bar indicates up-regulated pathways after *E2f4* knockdown, (*n* = 3). Data were analyzed by one-way ANOVA (I, K and L) and *t*-test (B, D, E and F). Error bars denote for the SD. ****P < 0.0001; ***P < 0.001; **P < 0.01; *P < 0.05

To explore the effects of E2F4 in RS, we overexpressed *E2f4* using a lentivirus system in pre-senescence mouse skin fibroblasts (P4), in which *E2f4* was expressed after doxycycline treatment (Fig. 5A). In this system, significantly increased *E2f4* expression was observed when compared with control (Fig. 5B and 5C). Moreover, the expression level of *E2f4* was correlated with the amount of lentivirus added (Fig. S5D). Interestingly, relatively higher amounts of lentivirus (MOI = 8 and MOI = 12) decreased cellular density and EdU percentage when compared with control (Fig. S5E and S5F). In comparison, cellular density and proliferative capacity were optimized with a lower MOI of 4, and this condition was used for further examination.

Compared with control, we found cells with *E2f4* overexpression had significantly decreased SA-β-Gal activity, smaller nuclear size and increased proliferative capacity (Fig. 5D–5F). Consistent with the EdU staining results, the transcriptional analysis showed that the expression of cell cycle-related genes was significantly increased by *E2f4* overexpression (Figs. 5G, 5H and S5G). In contrast, negative regulators of cell cycle, including *Cdkn2a* and *Cdkn2b*, showed decreased expression when compared with control (Fig. 5H), which highlighted the regulatory role of E2F4 in proliferation. RRBS analysis was performed to examine changes in DNA methylation after *E2f4* overexpression. Notably, some RS-associated DMSs were reversed after overexpression of *E2f4* (Fig. S5H).

In parallel, we also knocked down *E2f4* in proliferating mouse skin fibroblasts (P3) with shRNA (Fig. 5I and 5J). Consistent with the results of *E2f4* overexpression, knockdown of *E2f4* significantly decreased EdU positive percentage and increased nuclear size (Fig. 5K and 5L). Transcriptome analysis after knockdown of *E2f4* showed decreased expression of genes related to cell cycle regulation, and increased expression of genes related to inflammatory response and P53 pathway (Figs. 5M, S5I and S5J).

Taken together, these results indicated that overexpression of *E2f4* attenuated some senescence signatures, especially RS-associated reduction of proliferative capacity. Interestingly, we also found that overexpression of *E2f4* reversed some RS-associated methylation changes. These results highlighted the regulatory effects of E2F4 on RS phenotypes.

### Knockdown of *Tead1* and *Atf3* partially attenuated senescence-associated signatures

**Figure 6:**
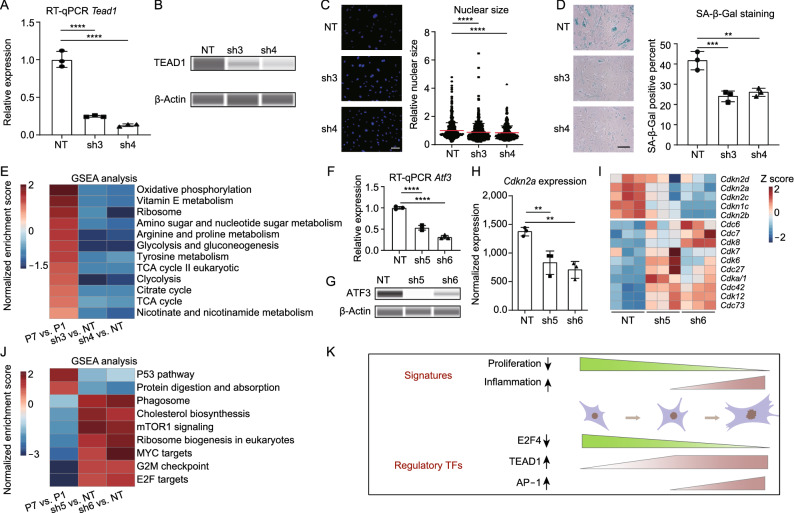
**Knockdown of Tead1 and Atf3 partially attenuated senescence-associated signatures**. (A) RT-qPCR detecting *Tead1* expression after knockdown with shRNAs, (*n* = 3). (B) Immunoblot showing the change in TEAD1 protein level after knockdown. (C) Scatter plot showing nuclear size distribution after *Tead*1 knockdown based on nuclear staining. Nuclear size was calculated using ImageJ, (*n* = 3). (D) Change in SA-β-Gal activity after *Tead1* knockdown. SA-β-Gal positive percentage was estimated based on 3 different views, and the point indicates the average value, (*n* = 3). Scale bar = 200 μm. (E) GSEA showing changes in RS-associated pathways related to cellular metabolism after *Tead1* knockdown. The color represents normalized enrichment score compared with control, (*n* = 3). (F) RT-qPCR detecting the change in *Atf3* expression after shRNA-mediated knockdown of *Atf3*, (*n* = 3). (G) Immunoblot showing the change in ATF3 protein level after knockdown. (H) Bar plot showing normalized expression of *Cdkn2a* after knockdown of *Atf3*, (*n* = 3). (I) Heatmap showing changes of selected cell cycle-related genes after knockdown of *Atf3*, (*n* = 3). (J) GSEA showing changes in selected RS-associated pathways after *Atf3* knockdown. The color represents normalized enrichment score compared with control, (*n* = 3). (K) Model showing the changes in RS-associated signatures (up) and regulatory TFs (down). Data were analyzed by one-way ANOVA (A, C, D, F and H). Error bars denote for the SD. ****P  < 0.0001; ***P < 0.001; **P < 0.01

TEAD1 was identified as a potential regulatory factor of RS in our previous analysis (Fig. 4A, 4D and 4F). To assess the role of TEAD1 in RS, we knocked down *Tead1* in mouse skin fibroblasts with shRNA (Figs. 6A and 6B). Interestingly, significantly decreased nuclear size and SA-β-Gal activity were observed after knockdown of *Tead1* (Fig. 6C and 6D). Transcriptome analysis after *Tead1* knockdown showed that some RS-associated changes in metabolism were altered, including decreased oxidative phosphorylation and citrate cycle (Fig. 6E).

Moreover, our analysis also identified ATF3, one member from AP-1 superfamily TFs, as a potential regulator of RS. Notably, we found more genes were regulated by ATF3 at senescent cells than proliferating cells using CUT&Tag analysis (Fig. S6A). Importantly, some typical senescence-related pathways were enriched in ATF3-regulated genes, such as the apoptosis process and cellular response to DNA damage stimulus (Fig. S6B).

To examine the role of ATF3 in RS, we knocked down *Atf3* in pre-senescent mouse skin fibroblasts (Fig. 6F and 6G). Consistent with the results from a previous study (Zhang et al., 2021), we found that knockdown of *Atf3* decreased *Cdkn2a* (*p16*) expression significantly (Fig. 6H). In line with this, some other negative regulators of cell cycle, including *Cdkn2c* and *Cdkn2d*, decreased expression after knockdown of *Atf3*. And increased expression of some cell cycle-related genes, including *Cdc6* and *Cdk8*, was observed when compared with control (Fig. 6I). In addition, GSEA analysis showed that some activated pathways in RS, including P53 pathway, were decreased after *Atf3* knockdown, and some cell cycle-related pathways, including G2M checkpoint and E2F targets, were found to increase after *Atf3* knockdown (Fig. 6J).

Collectively, our results demonstrated that knockdown of *Tead1* and *Atf3* attenuated some senescence-associated signatures, highlighting the potential regulatory roles of TEAD1 and AP-1 in RS.

## DISCUSSION

In this study, we employed integrative analysis of RS, including analysis of RNA-seq, RRBS and ATAC-seq datasets, and revealed that increased inflammation at late senescence and a gradual decline in proliferative capacity were significant signatures of RS in the transcriptome and epigenome. Moreover, we identified E2F4, TEAD1 and AP-1 as regulatory TFs of RS through integrative analysis and genetic manipulations (Fig. 6K).

In our study, we revealed transcriptional and epigenetic dynamics by making use of recent advances in multi-omics analysis, including time resolved RNA-seq, RRBS and ATAC-seq. Strong correlation between RS-associated epigenetic and transcriptional changes were observed (Fig. 3D). Importantly, we found conserved biological processes changed in both transcriptome and epigenome through integrative analysis. Among them, up-regulation of inflammatory response at late senescence and a gradual loss of replicative capacity were the most significant (Figs. 1E–G, 2E and 3E), which were consistent with previous studies (Coppé et al., 2008; Purcell et al., 2014; Hernandez-Segura et al., 2018). Importantly, consistent changes in transcriptional and epigenetic dynamics underlying these signatures were systematically described here. Moreover, in our study, a series of conservatively changed TFs in both transcriptome and epigenome were identified as potential regulators. These results highlight the advantages of time-resolved multi-omics analysis in investigating complex biological process like RS.

In our study, we identified E2F4 as a positive regulator of RS, which plays a role in regulating the senescence program, especially change in proliferative capacity. Through TF enrichment analysis based on transcriptome, we found E2F4 was enriched in RS-associated down regulated genes, and ATAC-seq analysis showed gradually decreased activity of E2F4 with passaging (Figs. 1D and 4C). Through genetic manipulations, we found that overexpression of *E2f4* increased cellular proliferative capacity and attenuated RS-associated changes in SA-β-Gal activity and nuclear size (Fig. 5D–F). Notably, our results also demonstrated that overexpression of *E2f4* reversed some RS-associated methylome signatures (Fig. S5H). We speculated that the epigenetic regulatory effects of E2F4 might be due to its ability to reshape the epigenome as a pioneer factor. Importantly, we found that overexpression of *E2f4* with a relatively higher copy numbers (MOI ≥ 8) repressed cellular proliferation rather than promoting it (Fig. S5E and S5F). This result indicated that the proliferation promoting effect of E2F4 was dose-related. In parallel, knockdown of *E2f4* in proliferating cells decreased cell proliferating capacity and induced other senescence phenotypes (Figs. 5I–M, S5I and S5J). E2F family TFs are key determinants of cell cycle process. As a member of E2F family, the E2F4 plays an important role in regulating cellular proliferation and development (Humbert et al., 2000; Hsu and Sage, 2016; Hsu et al., 2019). In addition, previous work demonstrated that reduction of E2F/DP activity caused cell cycle arrest, repressed DNA damage repair, and induced senescence-like phenotypes (Maehara et al., 2005; Collin et al., 2018), suggesting that E2F4 is a key regulatory TF affecting the RS program.

In addition, we also identified TEAD1 and AP-1 as negative regulators of RS affecting senescence phenotypes. The gene expression and TF activity of TEAD1 and AP-1 increased with passaging, and both of them were identified as pioneer factors of RS (Fig. 4G and 4F). However, the changes of TEAD1 occurred at the early stage of passaging. Unlike TEAD1, activation of AP-1 mainly occurred at late senescence (Fig. 4C and 4G). The heterochronous activation of these two TFs reflected the complexity of RS regulation. Through genetic manipulations, we found that knockdown of *Tead1* attenuated RS-associated changes in nuclear size and SA-β-Gal activity (Fig. 6C and 6D), and partially altered RS-associated transcriptional changes (Fig. 6E). The upregulation of TEAD family TFs during RS in human fibroblasts was reported recently (Chan et al., 2021), suggesting the role of TEAD1 in RS is species conserved. TEAD family TFs can interact with YAP, and are involved in many biological processes, including regulation of development and proliferation (Marti et al., 2015; Yu et al., 2015). Recently, YAP pathway was reported to be a positive regulator of PAI-1, one key modulator and marker of senescence and aging (Eren et al., 2014; Vaughan et al., 2017; Marquard et al., 2020). And Inhibition of YAP/TEAD could prevent senescence-like dormancy in cancer cells (Kurppa et al., 2020), suggesting potential regulatory roles of YAP/TEAD in senescence.

Through genetic manipulations, we also found that knockdown of *Atf3*, one member of AP-1 superfamily, decreased expression of *Cdkn2a* (*p16*) in senescent mouse fibroblasts (Fig. 6H), in agreement with previous work (Zhang et al., 2021). In addition, part of RS-associated transcriptional signatures related to cell cycle regulation was altered by *Aft3* knockdown (Fig. 6I and 6J). AP-1 is a dimeric complex that is composed of members from the JUN, ATF and MAF families (Shaulian and Karin, 2002). Recently, AP-1 was found to act as a pioneer factor in oncogene-induced senescence, and regulated senescence-associated inflammatory response (Martínez-Zamudio et al., 2020), indicating that AP-1 could be a conserved target for anti-senescence interventions.

Overall, our study demonstrated that RS was accompanied by significant transcriptome and epigenome changes using multi-omics analysis. By genetic manipulations, we revealed that E2F4, TEAD1 and AP-1 could affect RS-associated signatures as regulatory TFs, providing a set of novel targets for senescence and aging interventions. Nevertheless, our study has several limitations. We identified regulatory TFs of RS, however, the mechanisms underlying the functions remain unclear. In the future, more research needs to be employed to fill the gap of knowledge making use of recent advances in technologies. In addition, the regulatory relationship between these TFs and the pathways regulating their expression and activity should be explored in future studies. According to a previous study, RS *in vitro* could have a conserved mechanism with aging *in vivo* (Wagner et al., 2016). However, in this study, the regulatory effects of these identified TFs in aging were not explored. Considering the heterogeneity and complexity of aging *in vivo*, the regulatory effects of E2F4, TEAD1 and AP-1 in aging needed to be addressed with optimized experimental designs in the future. In addition, given that single TF has limited effects in regulating the senescence program, experiments will be carried out to explore the effects of TF combinations in anti-senescence and anti-aging.

## Material and methods

### Cell culture

Skin fibroblasts isolated from 2-month-old female C57BL/6 mice and fibroblasts from newborn mice (NBF) were cultured in high-glucose DMEM (Gibco) supplemented with 10% fetal bovine serum (FBS, VISTECH), 1% penicillin-streptomycin (Gibco) and 0.01% Plasmocin (InvivoGen). All cells were maintained at 37 °C in a humidified incubator with 5% CO_2_. When fibroblasts attained 80%–90% density, usually in 3 or 4 days, they were passaged at a ratio of 1:3 in 10 cm dishes and then cultured for different usages. Skin fibroblasts from 2-month-old female mice were used to prepare the time series multi-omics samples. For genetic manipulation, both skin fibroblasts from 2-month-old mice and NBF were used.

### RNA isolation and RNA-seq library preparation

TRIzol reagent (Invitrogen) was used to extract total RNA according to the manufacturer’s instructions. Subsequently, the RNA samples were sent to Novogene for subsequent cDNA library construction and Illumina sequencing with paired-end reads on a Novaseq PE150 system.

### DNA isolation and RRBS library preparation

Total DNA was extracted from cells using the MasterPure^TM^ Complete DNA & RNA Purification Kit (Cat.No. MC85200). The integrity of DNA was examined with an Agilent 2100 instrument. Subsequently, the DNA were sent to Novogene for subsequent RRBS library construction and sequencing with paired-ends on Novaseq PE150.

### ATAC-seq library preparation

ATAC-seq library for cells were prepared according to the library construction protocol as previously described (Corces et al., 2017). Briefly, after trypsin digestion, 50,000 viable cells were pelleted at 500 ×*g* at 4 °C for 5 min in a fixed angle centrifuge. To extract the nuclei, 50 µL cold lysis buffer (10 mmol/L Tris-HCl at pH 7.4, 10 mmol/L NaCl, 3 mmol/L MgCl_2_, 0.1% NP40, 0.1% Tween 20 and 0.01% digitonin) was added and incubated on ice for 3 min. After washing out the lysis buffer with wash buffer (10 mmol/L Tris-HCl at pH 7.4, 10 mmol/L NaCl, 3 mmol/L MgCl_2_, and 0.1% Tween 20), the nuclei were pelleted at 500 ×*g* for 10 min at 4 °C in a fixed angle centrifuge. After carefully removing the supernatant, the transposition was performed by resuspending the nuclei pellet in 50 µL of Transposition Mix containing 1× TD Buffer (20 mmol/L Tris-HCl pH at 7.6, 10 mol/L MgCl_2_ and 20% dimethyl formamide) and 2.5 µL Tn5 (Vazyme, TD501) for 30 min at 37 °C. Then, DNA was extracted with 2× DNA binding beads (Vazyme, N411-01). Libraries were produced by PCR, for which, the cycle numbers were determined by qPCR according to a previous publication (Buenrostro et al., 2015). Purify the final PCR reaction using 0.5–1.2× DNA binding beads. Library quality was assessed with an Agilent Bioanalyzer 2100, and paired end sequencing was performed with Illumina Hiseq PE150. Typically, 40–70 million high quality reads per library were required.

### CUT&Tag library preparation

CUT&Tag was performed with Hyperactive In-Site ChIP Library Prep Kit (Vazyme Biotech, TD902). In brief, 60,000 cells were incubated with 10 μL pre-washed ConA beads for 10 min. Primary antibody (E2F4, Proteintech, 10923-1-AP, 1:50; ATF3, abcam, ab207434, 1:50) was added and incubated for 2 h at room temperature. After washing twice with dig-wash buffer, Secondary antibody was added and incubated at room temperature for 45 min. After washing twice with dig-wash buffer, 0.58 μL pG-Tn5 was added with 100 μL dig-300 buffer. Samples were incubated at room temperature for 1 h and then washed twice with dig-wash buffer. 300 μL tagmentation buffer was added and incubated at 37 °C for 1 h. The reaction was stopped with 10 μL 0.5 mol/L EDTA, 3 μL 10% SDS and 2.5 μL 20 mg/mL Proteinase K. After extraction with phenol-chloroform and ethanol precipitation, PCR was performed to amplify the libraries using 16 cycles. All libraries were sequenced by Illumina Hiseq PE150.

### RT-qPCR

Total RNA was extracted with TRIzol reagent according to the manufacturer’s instructions (SIGMA). RNA was reversed-transcribed to generate cDNA using All-in-One Supermix (TransGen Biotech Co., Ltd.). qPCR was performed with KAPA SYBR FAST qPCR Master Mix (Roche, KK4602), and gene expression levels were normalized to *Gapdh*. All qPCR primers are listed in supplementary file 1.

### Experiment design for gene overexpression and knockdown

The potential transcription factors were overexpressed or knocked down in primary fibroblasts derived from newborn and 2-month-old mice. The changes in expression of target genes were confirmed by qPCR and Western blot. For overexpression experiment, doxycycline-induced gene expression system was used to fine tune the expression. 1 μg/mL doxycycline was added to the medium 4 days after transfection to avoid inducing gene expression when cells were under stress. And increasing MOIs were tested in preliminary overexpression experiment, and the optimized MOI was used for downstream analysis. Under the same culture condition, the cells that overexpressed EGFP were used as the control. To exclude the possibility of immortalization of primary fibroblasts, the cell growth was examined after removing doxycycline (data not shown). For knockdown experiment, two different shRNA sequences were used for each gene to avoid potential false positive results. In the experiments, P2 and P3 fibroblasts were used as proliferating cells, and P6 cells were used for senescence interventions.

### Overexpression and knockdown vector preparation

For *E2f4* overexpression vector construction, *E2f4* cDNA (NM_148952.1) was cloned into lentiviral vector FU-tet-on-P2A-EGFP (Addgene) through homologous recombination using ClonExpress II One Step Cloning Kit (Vazyme, C112). For gene knockdown, shRNA plasmids for the target genes were purchased from the MISSION® LentiPlex® Mouse Pooled shRNA Library (Merck). For each gene, at least two shRNA plasmids were prepared. All used shRNA sequence was listed in supplementary file 2.

### Lentiviral production, titration and viral transduction

HEK293T cells were used to produce lentivirus for overexpression and knockdown. When the cell density is about 80% confluence, they were co-transfected with proviral and packaging plasmids (pMDL-RPE, pRSV-REV, and pC1-VSVG) by polyethylenimine (PEI) methods as previously described (Joung et al., 2017). The medium was changed after 12–20 h, and the supernatant was collected every 24 h for 3 days. The total supernatant was filtrated using a 0.45 μm filter (MILLEX). The resulting viruses were tested for infectivity using HEK293T cells. For virus titration, shRNA viruses were tested by counting the cells remaining after puromycin selection (5 μmol/L for 24 h). The viruses used for overexpression were tested by flow cytometry using a GFP flag tag. For viral transduction, viruses were added to the cell culture medium with 8 µg/mL polybrene (Beyotime) supplementation, and the supernatant was removed 20 h later. For overexpression experiment, 1 μg/mL doxycycline was added to the medium 4 days after transfection, and GFP fluorescence was checked 24 h later. For knockdown experiments, 5 μmol/L puromycin was added to the medium until the non-infected control cells were all dead.

### Immunoblot analysis

Infected fibroblasts were collected and washed twice with pre-cold PBS and lysed in RIPA lysis buffer (Beyotime) containing 1× protease inhibitor (MCE) on ice for 30 min. Lysates were sonicated with Qsonica Q800R3 sonicator for 1 min at 40% amplitude with 5/7 s ON/OFF cycle followed by centrifugation at 15,000 rpm at 4 °C for 15 min. Protein concentrations in the cleared lysates were measured using BCA (Beyotime). Immunoblot analysis was performed by a simple western size-based protein assay (Protein Simple) following manufactures instructions. In brief, after loading of 750–1000 ng total protein onto each capillary, targeted protein level was identified using specific primary antibodies (E2F4, proteintech, 10923-1-AP; ATF3, abcam, ab207434; Tead1, abcam, ab133533; β-actin, abclonal, AC028; all 1:250 diluted). Chemiluminescence signals were analyzed using Compass software (Protein Simple).

### SA-β-Gal activity detection

After aspirating the media, the adherent cells were washed twice with PBS and fixed in SA-β galactosidase (SA-β-gal) staining fixing solution for 15 min at room temperature. Next, the cells were washed three times with PBS and incubated with 1.5 mL SA-β-gal staining solution (Beyotime Biotechnology, C0602) for 6-well plates for approximately 36–48 h at 37 °C. The plates were sealed with Parafilm to prevent evaporation of the staining medium. Finally, the incubated cells were washed with PBS and observed under a microscope to calculate the positive percentage.

### EdU staining for detection of cellular proliferating capacity and relative nuclear size calculation

EdU cell proliferation staining was performed using an EdU kit (Beyotime, C0071). Cells were seeded in 6-well plates and cultured in DMEM for 12–16 h. Subsequently, cells were incubated with EdU for 3 h according to the manufacturer's instructions. Next, the medium was aspirated and the cells were washed twice with PBS before fixing with paraformaldehyde. The cells were washed three times with washing buffer (PBS with 3% BSA) and permeated with 0.3% Triton X-100 for another 15 min. Next, the cells were washed three times with washing buffer, and incubated with the Click Reaction Mixture and Hoechst 33342 according to manufacturer's instructions. Cells stained with both green (or red) and blue were considered EdU-positive cells. Relative nuclear size was calculated using Image J v10.2 (NIH Image, Bethesda, MD, USA) on Hoechst-33342-stained images. Outlined nuclei were manually selected to ensure that only complete, well-separated nuclei were included in the study.

### Detection of SASP using Luminex assays

The relative concentration of selected cytokines were determined using Luminex with Mouse Cytokine Magnetic Beads Panel Kit (Millipore, MCYTOMAG-70K). The cell culture supernatant was collected and centrifuged at 5,000 ×*g* for 2 min to remove debris. Next, the relative concentration of selected cytokines were examined and calculated according to the manufacturer's instructions.

### RNA-seq data analysis

Sequencing adapters and low quality reads were removed from the raw RNA-seq reads with Trimmomatic (V0.39), with the main parameters set as follows: LEADING:3 TRAILING:3 SLIDINGWINDOW:4:15 MINLEN:26. The trimmed reads were then mapped to the mm10 mouse genome using Hisat2 (V2.2.1). Next, we used HTSeq-count (V0.12.4) to calculate the count of reads for each gene, with the main parameters set as: -f bam -s no -r pos. The reference genome was downloaded from the USCS Table Browser. To quantify gene expression, the read count for each gene was normalized using DESeq2 (V1.22.2). The differentially expressed genes from each group in comparison with P1 were filtered with following criteria: adjusted *P*-value < 0.05 and fold-change >2 or <0.5. All differentially expressed genes were divided into clusters using the pheatmap package, and genes that were monotonically expressed with passaging were identified as RS-associated genes.

### ChIP-seq analysis

The public ChIP-seq data for H3K4me1 and H3K27ac in replicative senescent mouse skin fibroblasts were obtained from GSE117210. Trimmomatic was used to trim reads with the parameters set as follows: LEADING:3 TRAILING:3 SLIDINGWINDOW:4:15 MINLEN:26. Bowtie2 (V2.3.5.1) was used to map the trimmed reads to the mm10 reference genome. Picard was used to remove duplicate reads with default parameters. Next, Macs2 (V2.2.7.1) was used for peak calling with the parameters set as follows: --broad -g mm -B --broad-cutoff 0.1. The enhancer regions were identified as those that were double-positive for H3K4me1 and H3K27ac peaks using bedtools (V2.29.2).

### ATAC-seq data analysis

The ATAC-seq data were pre-processed (trimmed, aligned, filtered and quality controlled) using the ATAC-seq pipeline from the Kundaje lab (https://github.com/ENCODE-DCC/atac-seq-pipeline). In the pipeline, the input JSON file included genomic data files, parameters for running the pipeline. Using the auto-detected adapter mode to trim the reads, we set the parameters to “-e 0.1 -m 5”. Bowtie2 was used to align the reads to the reference genome. The parameters for mapping were set as “-X2000 --mm –local”. After the alignment, duplicates were removed by Picard and only uniquely mapped alignments (MAPQ > 10) were kept. MACS2 was used for peak calling, and the parameters for MACS2 was set as “-g mm -B --shift -100 --extsize 200 --nomodel”. The “norrowpeak” files were used for downstream analysis. The peaks that appeared in more than 60% of the replicate samples in each group were kept for feature counts and subsequent analysis using DiffBind (V2.12.0). The read counts for each peak were normalized using DESeq2 (V1.22.2). Peaks that differed significantly between any groups were identified using DESeq2 with the following criteria: adjusted P-value < 0.05 and fold-change > 2 or < 0.5. All peaks with significant differences were divided into modules using the pheatmap package, and peaks that monotonically changed with passaging were identified as RS-associated peaks. Peak annotation was performed with HOMER (V4.10). And the peaks that overlapped with gene promoters (±1 kb region around the TSS) were identified as gene-regulating peaks.

### RRBS data analysis

Sequencing adapters and low-quality reads were removed from the raw sequencing reads using Trim Galore (V0.6.6) with the following parameters: --stringency 1 --length 2 -e 0.1 --rrbs. Bismark was used for alignment to the mm10 reference genome with default parameters. To calculate the methylation level of each cytosine, bismark_methylation_extractor was used with the parameters set as follows: --gzip --bedGraph. The correlation and PCA analyses were performed using Methylkit (V1.12.0) with default parameters. DMRs between any two groups were identified using DSS (V2.34.0) with the parameters set as follows: delta = 0.1, p.threshold = 0.01. The average DNA methylation level for each DMR was calculated with bedtools. All DMRs were clustered using the pheatmap package, and DMRs with monotonic DNA methylation changes with passaging were identified as RS-associated DMRs.

### CUT&Tag data analysis

Sequencing data was aligned to the mm10 reference genome using bowtie2 with the following parameters: --end-to-end --very-sensitive --no-mixed --no-discordant --phred33 -I 10 -X 700. Then, samtools and bedtools were used to transfer aligned files into bedgraph format file without removing duplicated reads. Peak calling was performed with SEACR (V1.3) and the parameters are: norm stringent. The conserved peaks between duplicate samples were kept using DiffBind for downstream analysis. HOMER (V4.10) was sued for peak annotation, Genes, whose promoters were overlapped with the identified peaks, were regarded as downstream genes of this TF.

### Identification of pioneer factors using PIQ

Pioneer factors were identified based on CD and the COI, which were calculated using PIQ (V1.3) with published code according to methods included in a recently published paper (Sherwood et al., 2014; Martínez-Zamudio et al., 2020). TFs with CD greater than 0.45 and a COI greater than 4 were defined as pioneer factors, TFs with CD greater than 0.45 and a COI less than 4 were defined as settler factors, and TFs with CD less than 0.45 were defined as migrator factors.

### TF enrichment analysis using RcisTarget

Potential regulatory TFs of genes from different clusters were identified via RcisTarget (V1.6.0), an R package identifying transcription factor binding motifs enriched on a list of genes or genomic regions, with default parameters.

### Identification of RS-associated DMS

Firstly, cytosines with monotonically increased methylation were filtered. Next, cytosines with a methylation change greater than 0.05 between adjacent passages in all 3 series of samples were identified as RS-associated increased DMSs. In total, 38 cytosines were identified. Using the same method, but with a lower threshold (0.03 between adjacent passages), 39 RS-associated decreased DMSs were identified.

### Motif enrichment analysis

Motif enrichment analysis was performed based on selected genome regions using HOMER (V4.10). For ATAC-seq peak-based motif analysis, the parameters were set as “-len 8,10,12,15 -p 8 -S 40 -keepOverlappingBg -bg” because some TFs were specifically activated in skin fibroblasts. All narrow peaks identified in the ATAC-seq data were used as background. For the DMR-based motif analysis, the parameters were set as follows: -len 8,10,12,15 -p 8 -S 40.

### TF footprint analysis based on ATAC-seq data

In the ATAC-seq method, the Tn5 enzyme recognizes and cleaves DNA in open regions, which causes enhanced alignment of reads in these regions. However, the presence of TFs binding to DNA prevents Tn5 from cleaving DNA at TF binding sites, leaving small regions, referred to as footprints, where the read coverage drops sharply within peak regions with high coverage (Li et al., 2019). For the TF footprint analysis, RGT-HINT (V0.13.0) footprinting was used with the parameters set as follows: --atac-seq --paired-end --organism=mm10.

### Classifying TFs as activators or repressors

TFs can activate or repress expression of downstream genes. DiffTF (V1.8) was used to classify TFs as activators or repressors by integrative analysis of RNA-seq and ATAC-seq data with default parameters as described previously (Berest et al., 2019).

### GSEA analysis

For GSEA analysis, hallmark gene sets (V7.4) were downloaded from MSigDB (http://www.gsea-msigdb.org/gsea/downloads.jsp). GSEA was performed with normalized expression data using python package GSEApy (0.10.2) with the parameters set as follows: cls=class_vector, method='signal_to_noise', min_size=5, max_size=5000. FDR q-value of 0.05 or less were considered significant.

## Supplementary Information

Below is the link to the electronic supplementary material.

Supplementary file1 (PDF 2827 kb)
