# Supplementary material for: Unveiling E2F4, TEAD1 and AP-1 as regulatory transcription factors of the replicative senescence program by multi-omics analysis
Source: Protein Cell. 2022 Jan 12;13(10):742–59. doi: 10.1007/s13238-021-00894-z (PMC9233726; doi:10.1007/s13238-021-00894-z)
Supplement: Supplementary file 1 — Supplementary file1 (PDF 2827 kb) [file 13238_2021_894_MOESM1_ESM.pdf]

Supplementary file 1

Primer name	Sequence (5’-3’)
Gapdh-F	GGCAAATTCAACGGCACAGT
Gapdh-R	GTCTCGCTCCTGGAAGATGG
E2f4-F	CTCACCACCAAGTTCGTGTC
E2f4-R	TCTCGATCAGACCGATGCCTT
Tead1-F	AAGCTGAAGGTAACAAGCATGG
Tead1-R	GCTGACGTAGGCTCAAACCC
Atf3-F	GAGGATTTTGCTAACCTGACACC
Atf3-R	TTGACGGTAACTGACTCCAGC
Cdkn2a-F	GCCCAACGCCCCGAACTCTTTC
Cdkn2a-R	GCGACGTTCCCAGCGGTACACA

Supplementary file 2

ShRNA	Target	Sequence (5’ to 3’)
sh1	E2f4	CCGGGAAGAACTGATGTCTTCAGAACTCGAGTTCTGAAGACATCAGTTCTTCTTTTTG
sh2	E2f4	CCGGCATCGGTCTGATCGAGAAGAACTCGAGTTCTTCTCGATCAGACCGATGTTTTTG
sh3	Tead1	CCGGCGCTCGCCAATGTGTGAATATCTCGAGATATTCACACATTGGCGAGCGTTTTTG
sh4	Tead1	CCGGGCTCGCCAATGTGTGAATATACTCGAGTATATTCACACATTGGCGAGCTTTTTG
sh5	Atf3	CCGGGCATCCTTTGTCTCACCAATTCTCGAGAATTGGTGAGACAAAGGATGCTTTTTG
sh6	Atf3	CCGGACCTCTTTATCCAACAGATAACTCGAGTTATCTGTTGGATAAAGAGGTTTTTTG
NT	shControl	CCGGGCGCGATAGCGCTAATAATTTCTCGAGAAATTATTAGCGCTATCGCGCTTTTT

Supplementary figure 1

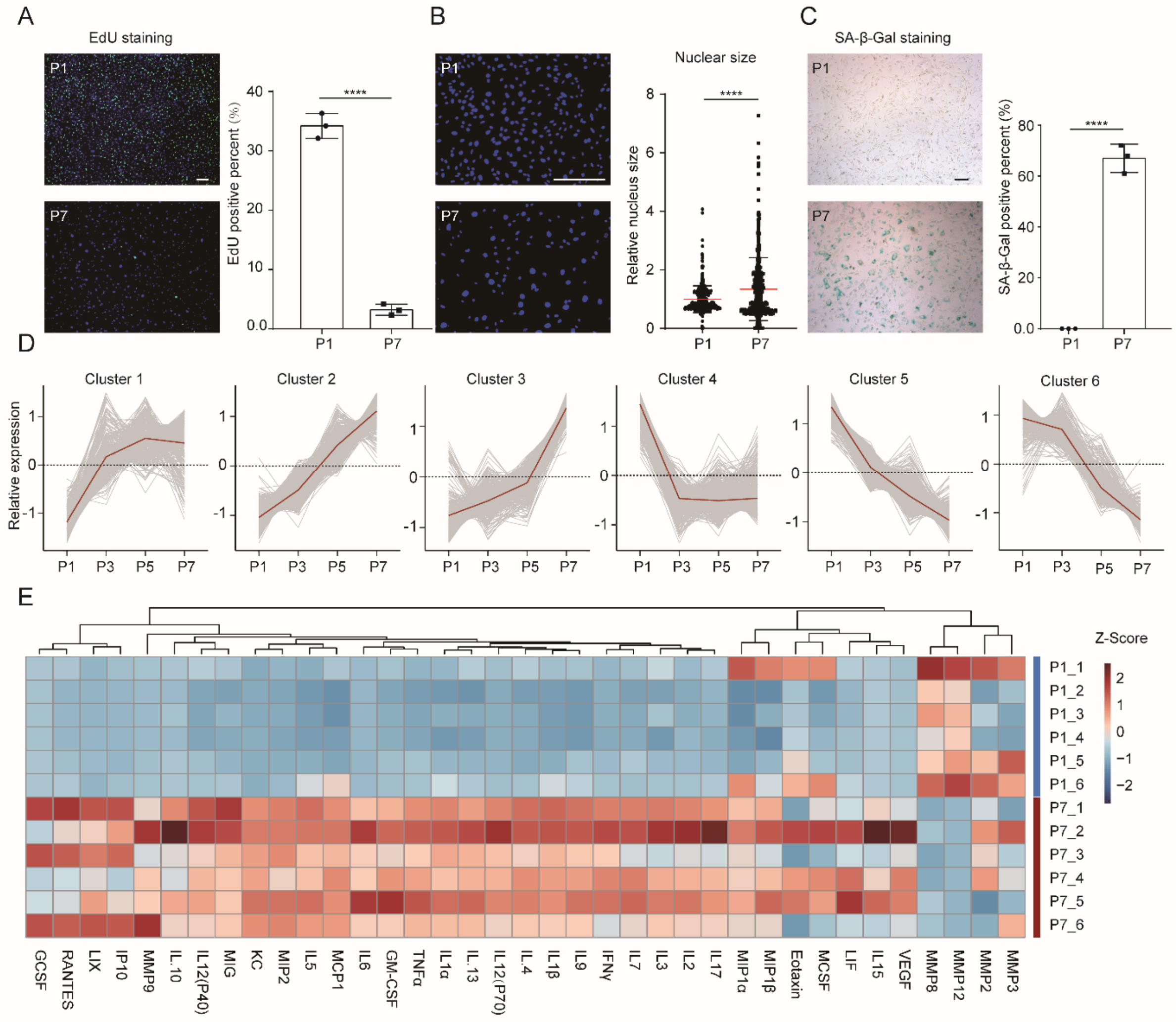

Supplementary Figure1. Identification of senescence based on senescence associated hallmarks.

(A) EdU staining showing the change in replicative capacity after senescence. Representative images of EdU staining of non-senescent (top) and replication-induced senescent fibroblasts (bottom). Green dots indicated EdU positive cells, and the EdU positive percentage was calculated using ImageJ, (n=3). Scale bar =200 μm. (B) Estimation of change in nuclear size after senescence based on nuclear staining, (n=3). Representative images of nuclear staining of non-senescent (top) and replication-induced senescent fibroblasts (bottom). Scale bar = 200 μm. (C) The change in SA-β-Gal activity after senescence. Representative images of non-senescent (top) and replication-induced senescent fibroblasts (bottom), (n=3). Scale bar = 200 μm. (D) Expression dynamics of genes in each cluster during RS. Each grey line represents one gene in the corresponding cluster, and the dark red line represents the average expression of all genes in the cluster. The expression of genes were normalized by Z score. (E) Heatmap showing changes in SASP concentration with RS in culture supernatant using Luminex, (n=6).

Data were analyzed by *t* test (A, B and C). Error bars denote for the SD. **** *P*<0.0001.

Supplementary figure 2

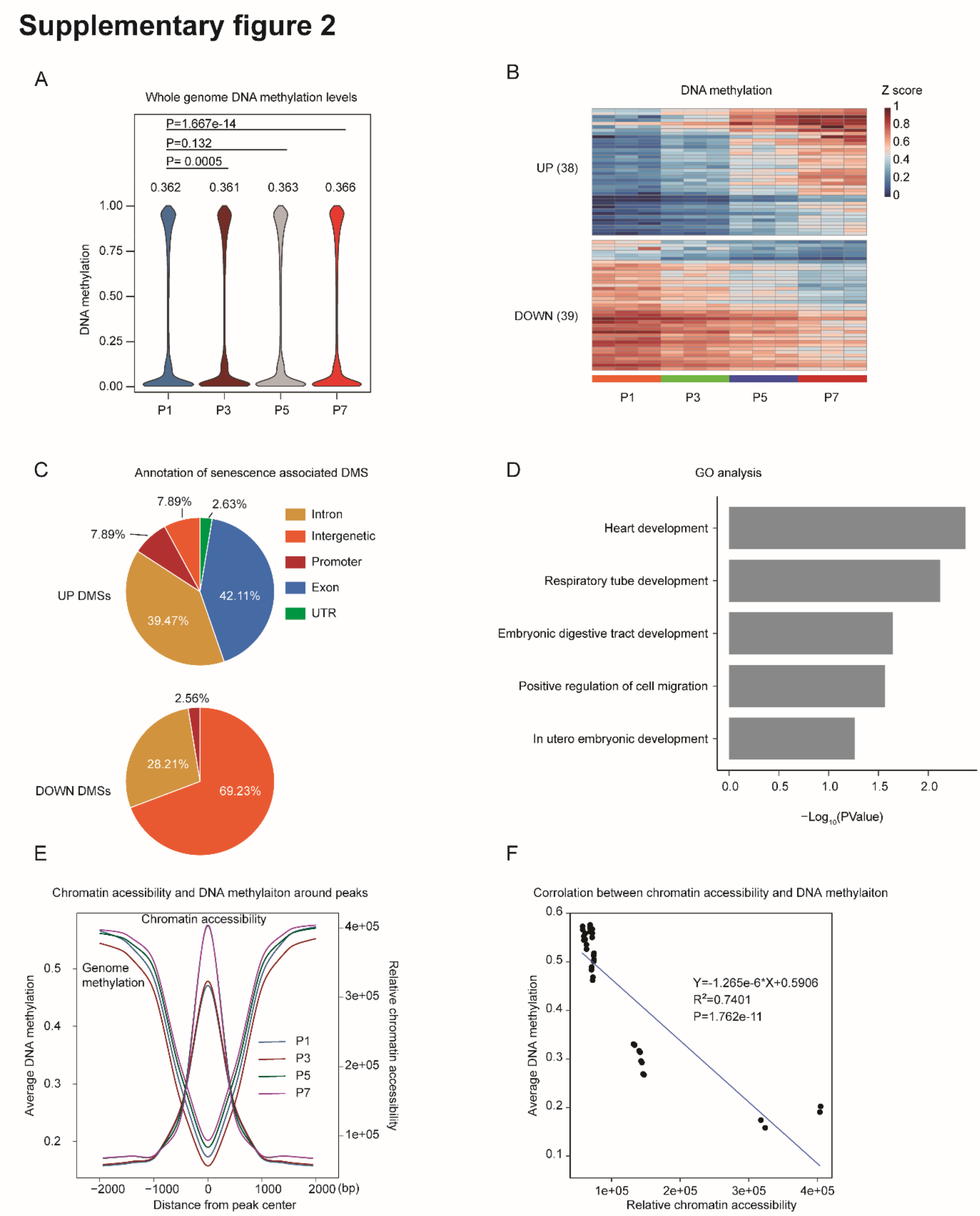

Supplementary Figure 2. Identification of replicative senescence-associated signatures in genetic methylome.

(A) Violin plots summarizing the distribution of DNA methylation across the entire genome for each group. The average DNA methylation level and significance compared with P1 is labeled at the top, (n=3). (B) Heatmap showing changes in DNA methylation for RS-associated DMSs with passaging, including 38 hypermethylated DMSs (UP) and 39 hypomethylated DMSs (DOWN). (C) Pie charts showing the locations of DMSs on the genome for hypermethylated DMSs and hypomethylated DMSs. (D) Biological process GO enrichment analysis based on genes related RS-associated hypermethylated DMSs. The genes whose gene bodies included RS-associated DMSs were identified as DMS-related genes. (E) Line plot showing the dynamics of DNA methylation and relative chromatin accessibility around the center of peaks of ATAC-seq. The average DNA methylation and relative chromatin accessibility were calculated in every bin (500 bp length) around the center of ATAC-seq peaks. The relative chromatin accessibility was normalized based on

total reads number. (F) Regression plot showing the correlation between the chromatin accessibility and DNA methylation. Each dot indicated the value of the average DNA methylation and relative chromatin accessibility of each bin. $R^2 = 0.7401$, $P = 1.762e-11$.

Supplementary figure 3

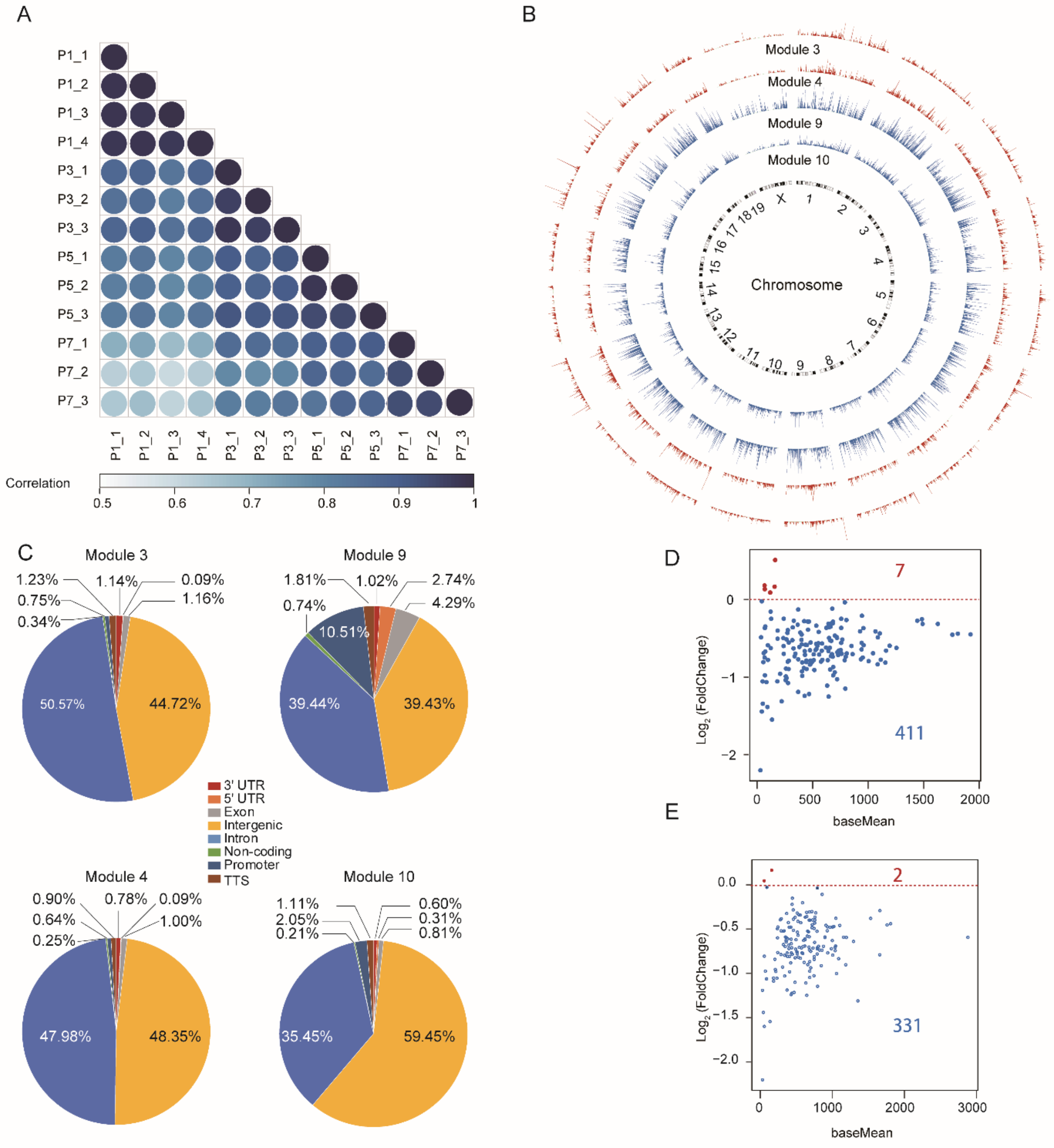

Supplementary Figure3. Chromatin accessibility dynamics of replicative senescence.

(A) Heatmap showing Pearson's correlations between samples based on ATAC-seq (n=4 for P1, n=3 for P3, P5 and P7). (B) Circos plot showing the distribution and accessibility of peaks from module 3, module 4 (red), module 9 and module 10 (blue) on the genome. (C) Pie charts showing the distributions of peaks from module 3, module 4, module 9 and module 10 on the genome. (D) MA plot of senescent cells (P7) and proliferating cells (P3) showing the log2 ratios of peak accessibility versus mean-normalized read counts for peaks related to G2M checkpoint-associated genes (the gene set was downloaded from MSigDB). (E) MA plot of senescent cells (P7) and proliferating cells (P3) showing the log2 ratios of peak accessibility versus mean-normalized read counts for peaks related to E2F target genes (the gene set was downloaded from MSigDB).

Supplementary figure 4

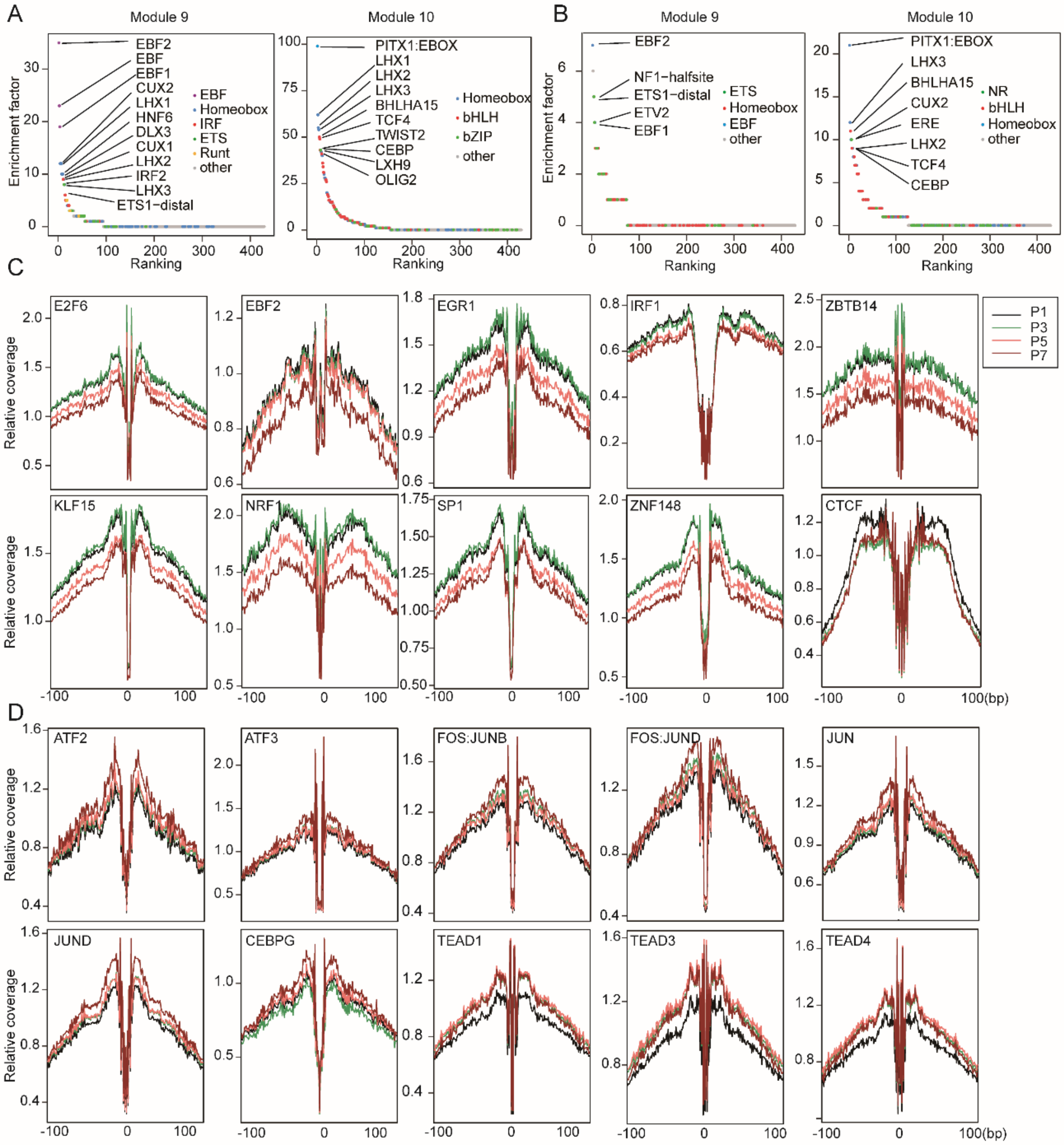

Supplementary Figure4. Identification of replicative senescence-associated regulatory TFs.

(A) Ranking of motifs enriched in RS-associated peaks from module 9 and module 10. (B) Ranking of motifs enriched in RS-associated peaks located in enhancer regions from module 9 and module 10. The colors of the points represent different TF families. (C-D) Transcription factor footprinting in chromatin-accessible regions based on ATAC-seq. The mean ATAC-normalized coverage averaging the forward and reverse strand within 100 bp upstream and downstream of the corresponding transcription factor motif sites is shown. The ATAC normalized coverage dynamics from P1 (black line), P3 (green line), P5 (orange line) and P7 (dark red line) are shown. (C) RS-associated up-regulated TFs. (D) RS-associated down-regulated TFs.

Supplementary figure 5

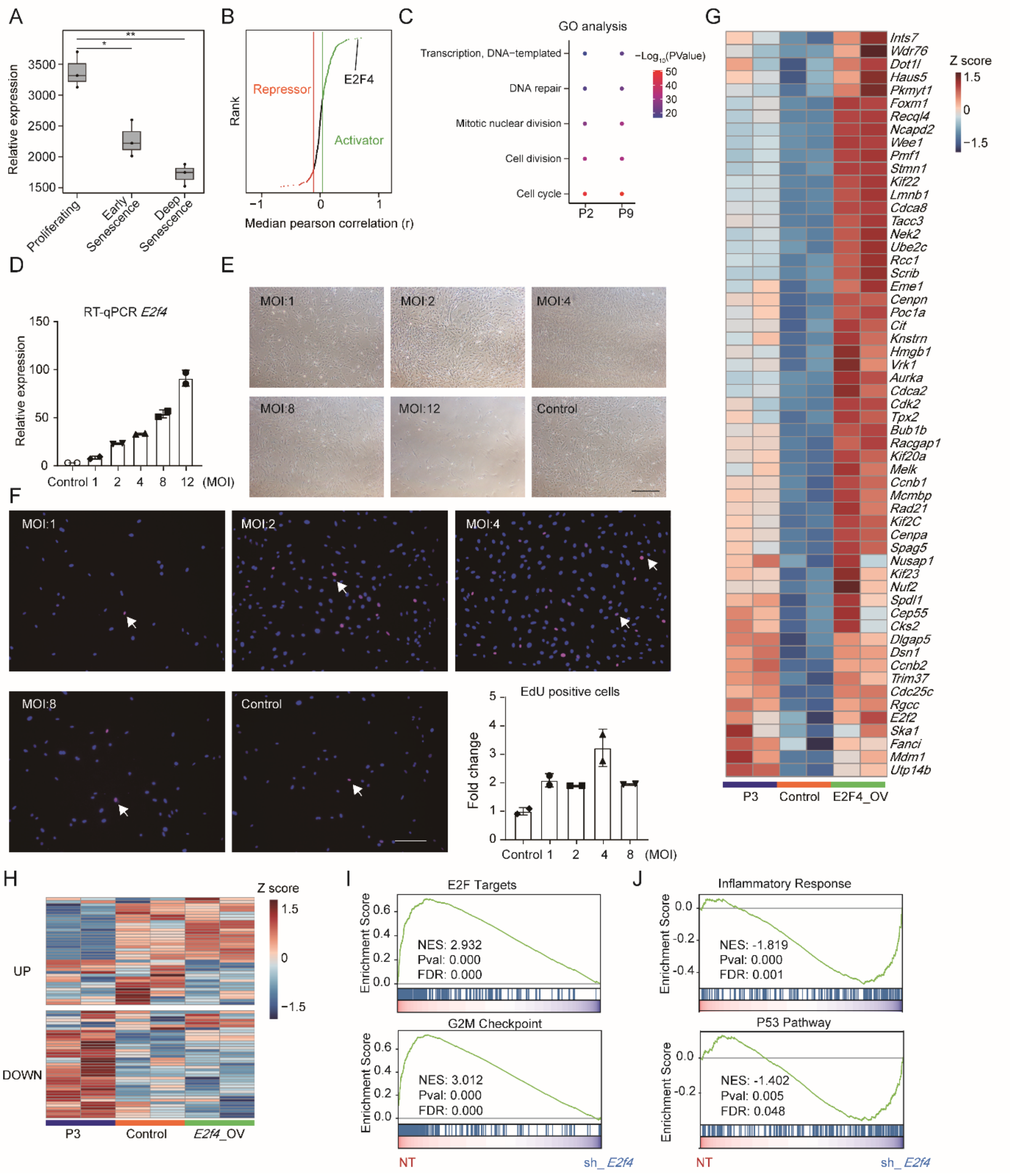

Supplementary Figure5. E2F4 affects replicative senescence-associated phenotypes as a regulatory TF.

(A) Bar plot showing the change in *E2F4* expression with passaging in human ES-derived lung fibroblasts (GSE109700), (n=3). (B) Classifying TFs as activators or repressors based on ATAC-seq and RNA-seq using DiffTF. E2F4 was identified as an activator of downstream genes (P<0.001). (C) Biological process GO analysis based on E2F4-regulated genes, which were identified via CUT&Tag analysis. Only the top 5 most significantly enriched terms in P2 and P9 cells were shown, (n=2). (D) RT-qPCR detecting *E2f4* expression after lentivirus-mediated over-expression with different MOIs (n=2). (E) Light field images showing the change in cellular density after *E2f4* overexpression with different MOIs. Scale bar = 200 μm. (F) EdU staining showing the change in proliferative capacity after *E2f4* overexpression with different MOIs. Blue dots indicate nuclear staining, and red dots indicate EdU-positive cells (white arrows), (n=2). Scale bar = 200 μm. (G) Heatmap showing changes in the expression levels of cell cycle-related genes based on RNA-seq, (n=2). (H) Heatmap showing changes in DNA methylation of identified RS-associated DMSs after *E2f4*

overexpression, (n=2). (I-J) GSEA showing the change in RS-associated pathways related to cell cycle (I) and inflammation (J), (n=3).

Data were analyzed by one-way ANOVA (A). Error bars denote for the SD. ** $P<0.01$; * $P<0.05$.

Supplementary figure 6

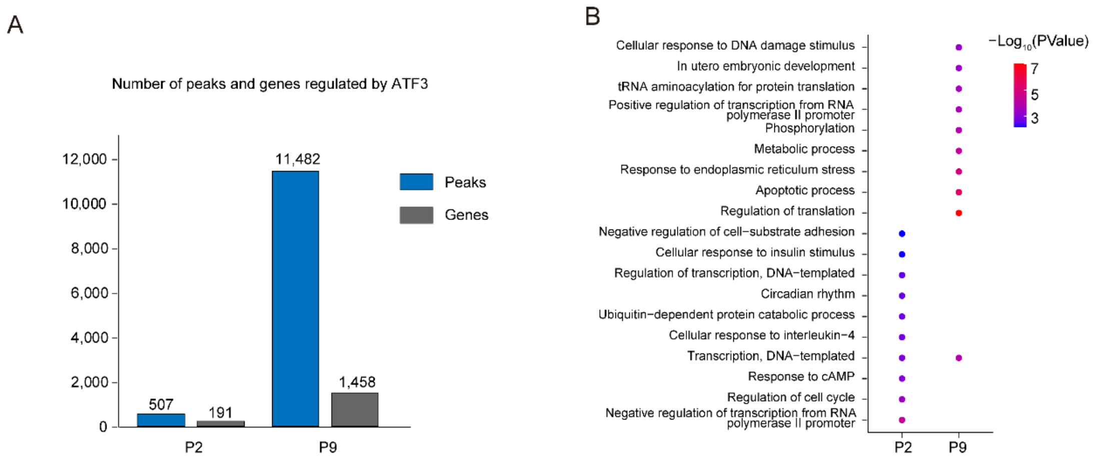

Supplementary Figure6. Some RS-associated pathways were under regulation of activated ATF3 during senescence.

(A) Bar plot showing the number of peaks identified via ATF3 CUT&Tag in proliferating (P2) and senescent cells (P9) (blue bar), and the number of genes under regulation of ATF3 (grey bar). The genes whose promoters were overlapped with identified ATF3 CUT&Tag peaks were regarded as under the regulation of ATF3, (n=2). (B) Biological process GO enrichment analysis based on ATF3-regulated genes in proliferating (P2) and senescent cells (P9). Top 10 most significantly enriched terms were shown.
